# Temporal trends in Group B *Streptococcus* colonization, serotype distribution, and antimicrobial resistance among pregnant women in the Brazilian Amazon across the COVID-19 pandemic, 2018–2023

**DOI:** 10.1007/s10123-026-00832-1

**Published:** 2026-04-25

**Authors:** Anjo Gabriel Carvalho, Mayra Gyovana Leite Belém, Erilene de Lima Sinos, Marcos Eduardo Passos da Silva, Renata Santos Rodrigues, Izabelly Vitória Gotara Ramos, Gil Guibson Mota Amaral, Luccas Manoel de Melo Suica, Valcimar Ferreira Batista, Núcia Cristiane da Silva Lima, Mariana Delfino Rodrigues, Roger Lafontaine Mesquita Taborda, Najla Benevides Matos

**Affiliations:** 1 Postgraduate Program in Experimental Biology, Federal University of Rondônia, Porto Velho, Rondônia Brazil; 2 Laboratory of Microorganism Biology, Oswaldo Cruz Foundation, Porto Velho, Rondônia Brazil; 3 Center for Research in Tropical Medicine, Porto Velho, Rondônia Brazil; 4 Department of Nursing, Aparício Carvalho Integrated Colleges, Porto Velho, Rondônia Brazil

**Keywords:** *Streptococcus agalactiae*, Pregnant women, Antimicrobial resistance, Multidrug resistance, COVID-19 pandemic

## Abstract

**Purpose:**

Group B *Streptococcus* (GBS) is a leading cause of preterm birth and neonatal infection. The COVID-19 pandemic substantially altered healthcare access, antibiotic use, and pathogen circulation. However, its influence on GBS remains poorly understood. This study evaluated temporal changes in GBS colonization, serotypes, and antimicrobial susceptibility among pregnant women in the Brazilian Amazon before, during, and after the COVID-19 pandemic.

**Methods:**

A total of 1,449 pregnant women at 35–37 weeks of gestation attending public health units in Porto Velho, Brazil, between February 2018 and November 2023 were included. Rectovaginal samples were cultured for GBS detection. Capsular serotyping and antimicrobial susceptibility testing were performed using standardized methods. Colonization prevalence, serotype distribution, and resistance profiles were compared across pre-pandemic, pandemic, and post-pandemic periods.

**Results:**

Overall GBS colonization was detected in 20.7% (300/1,449) of participants, with variation across periods **(**22.6% before the pandemic, 21% during, and 19% after). Serotype Ia predominated (39%), followed by serotypes V (15.8%), II (14.1%), Ib (11.9%), III (10.2%), IV (5.1%), and VI (4%). Serotype distribution varied across periods without statistically significant differences. Non-susceptibility profiles differed significantly among serotypes. All isolates were susceptible to penicillin, ampicillin, cefazolin, and vancomycin. Increased non-susceptibility was observed for macrolides and levofloxacin, with multidrug-resistant (MDR) isolates rising from 5.7% to 15.9% post-pandemic.

**Conclusion:**

Together, these findings demonstrate temporal variation in GBS colonization, serotype distribution, and antimicrobial resistance across pre-, during-, and post-pandemic periods. While colonization, serotype distribution, and β-lactam susceptibility remained stable, significant increases in non-susceptibility to macrolides and levofloxacin, along with higher proportions of MDR profiles, were observed in later periods. These results highlight the importance of continued surveillance and provide relevant epidemiological insights to inform vaccine implementation and public health strategies in the post-pandemic era.

**Supplementary Information:**

The online version contains supplementary material available at 10.1007/s10123-026-00832-1.

## Introduction

*Streptococcus agalactiae* (Group B *Streptococcus*, GBS) is a leading cause of infection during pregnancy, preterm birth, and neonatal disease, particularly in low- and middle-income countries (LMICs) (Gonçalves et al. 2022). GBS frequently colonizes the gastrointestinal and genitourinary tracts of healthy adults. In pregnant women, rectovaginal colonization during late gestation is the main risk factor for the development of invasive neonatal disease (Brokaw et al. 2021). Neonatal GBS disease is classified as early-onset disease (EOD) when it occurs in the first 7 days of life, typically presenting as sepsis or pneumonia, or late-onset disease (LOD), which occurs between 7 and 90 days of life and is commonly associated with meningitis and sepsis (Raabe and Shane 2019). It is estimated that an average of 18% of pregnant women worldwide are colonized with GBS (with regional variation ranging from 11% to 35%), resulting in 518,000 preterm births, 392,000 neonatal infections, and 91,000 deaths annually (Russell et al. 2017, WHO 2021).

GBS can be classified into 10 serotypes (Ia, Ib, II–IX) based on the expression of a sialic acid–rich capsular polysaccharide (CPS). CPS is also an important virulence factor that facilitates evasion of innate immunity, promotes adhesion, and invasion of host tissues (Brokaw et al. 2021). Serotypes differ in their virulence potential and their geographical or temporal distribution in both colonization and invasive disease. Globally, serotypes Ia, Ib, II, III, and V are the most common in the perinatal context (Russell et al. 2017, Seale et al. 2017). As one of the primary discriminatory markers of GBS, serotyping is widely used in epidemiological studies and remains the main target for GBS vaccine development. As vaccination efforts have advanced, monitoring serotype diversity has become increasingly important, as selective pressures may drive serotype replacement or favor the expansion of non-vaccine serotypes. So, serotype dynamics characterization across different epidemiological periods is critical for anticipating vaccine coverage and guiding long-term disease control strategies (Furfaro et al. 2018, Madhi et al. 2023).

Preventive strategies, particularly intrapartum antibiotic prophylaxis (IAP), have significantly reduced the burden of neonatal GBS disease in high-income countries (HICs), decreasing EOD incidence by 86%–89% among infant borns to colonized mothers (Verani et al. 2010, American College of Obstetricians and Gynecologists 2020). In LMICs, including Brazil, IAP implementation remains heterogeneous, with some guidelines recommending screening but no national consensus in place (Boureka et al. 2023). This regional variability in access to screening and prenatal care contributes to gaps in epidemiological surveillance and limits the ability to track temporal shifts in maternal colonization and antimicrobial resistance.

Although GBS remains highly susceptible to first-line β-lactams, resistance to macrolides, lincosamides, and other antimicrobial classes has continued to rise (Hayes et al. 2020, Gergova et al. 2024, Bostanghadiri et al. 2025). In 2019, GBS accounted for an estimated 100,000 to 250,000 antimicrobial resistance (AMR)-associated deaths globally, ranking tenth among bacterial pathogens and prompting its inclusion on the Centers for Disease Control and Prevention (CDC) antimicrobial resistance threat list and in the 2024 World Health Organization (WHO) Bacterial Priority Pathogens List (Centers for Disease Control and Prevention 2019, Sati et al. 2025). Given that AMR patterns vary across regions and evolve over time, sustained phenotypic and molecular surveillance is necessary to detect emerging resistance trends and inform treatment policies. This need is intensified in LMICs, where surveillance systems for GBS remain limited and fragmented.

The coronavirus disease 2019 (COVID-19) pandemic further altered the epidemiology of infectious diseases worldwide, contributing to accelerated AMR, shifting pathogen circulation, and modifying healthcare practices (Juan et al. 2021, Langford et al. 2023). Several countries reported increases in invasive and nosocomial GBS infections during the first pandemic year, often associated with multidrug-resistant (MDR) strains (Brueggemann et al. 2021, Shaw et al. 2023, Huang et al. 2023). However, despite the recognized impact of the pandemic on healthcare access and antibiotic consumption, data on maternal GBS colonization—particularly trends spanning pre-, peri-, and post-pandemic periods—remain scarce. A few studies have examined changes in colonization, serotype distribution, and AMR during the first pandemic year, and none have evaluated extended temporal trends (Costa et al. 2022, Serra et al. 2024). Therefore, evaluating colonization patterns across distinct pandemic phases represents an important opportunity to explore the stability of the maternal reservoir and the potential emergence of adaptive shifts in circulating strains.

In Brazil, the COVID-19 pandemic profoundly disrupted antenatal care, childbirth services, and postpartum follow-up, limiting timely access to prenatal screening (Orellana et al. 2022, Tenorio et al. 2022). As the prevention of neonatal GBS disease prevention depends on screening at the last trimester of pregnancy—and in the absence of a national screening or IAP policy—generating robust epidemiological data is essential to support public health planning, particularly in regions with limited resources and heterogeneous access to care, such as the Brazilian Amazon. Therefore, this study aims to evaluate and compare the prevalence of GBS colonization, serotype distribution, and antimicrobial susceptibility among isolates obtained from pregnant women in Porto Velho, Brazil, across three epidemiologically distinct periods: before, during, and after the COVID-19 pandemic.

## Materials and methods

### Study design and setting

A retrospective cross-sectional study was conducted between February 2018 and November 2023 in Porto Velho, the capital of the state of Rondônia. Located in the western portion of the north of Brazil, on the western edge of Amazon rainforest, Porto Velho had an estimated population of 460,434 inhabitants in 2022, according to the Brazilian Institute of Geography and Statistics (Instituto Brasileiro de Geografia e Estatística 2022). Eleven Basic Healthcare Units specialized in the care of low- and medium-risk pregnant women were selected, as well as the Integrated Mother and Child Center, a reference center responsible to monitor high-risk pregnancies.

### Study population

A total of 1,449 pregnant women between 35 and 37 weeks of gestation were included in the study. Participants were categorized according to three periods: February 2018 to February 2020 represented the pre–COVID-19 pandemic period (BC, *n* = 443); March 2020 to April 2023 represented the period during the COVID-19 pandemic (DC, *n* = 774); and samples collected from May 2023 to November 2023 represented the post–COVID-19 pandemic period (AC, *n* = 232). The post-pandemic period was defined based on the WHO declaration of the end of COVID-19 as a public health emergency on May 11, 2023.

### Ethical considerations

This study was approved by the Research Ethics Committee of the Research Center in Tropical Medicine (CEP/CEPEM) under approval number 76.812–329.812. Written informed consent was obtained from all participants.

### Data collection

A semi-structured questionnaire was used to collect data on age, marital status, ethnic background, education level, occupation, obstetric history, and sexual activity.

### Specimen collection

Rectal and vaginal secretion samples were collected by healthcare professionals during routine prenatal outpatient visits using sterile swabs placed in Stuart transport medium (CRAL, São Paulo, Brazil). The collected material was transported to the Microbiology Laboratory of the Oswaldo Cruz Foundation of Rondônia (FIOCRUZ) and processed in 24 h after collection, following American Society for Microbiology recommendations (American Society for Microbiology 2021).

### Detection and isolation of GBS from rectovaginal samples

Swabs were inoculated into Todd-Hewitt broth (KASVI, Paraná, Brazil) supplemented with gentamicin (8 µg/mL; Interlab, São Paulo, Brazil) and nalidixic acid (15 µg/mL; Sigma-Aldrich, Missouri, USA), then incubated at 37 °C with 5% CO₂ for 24 h. Bacterial DNA was extracted from the enriched cultures using the phenol–chloroform method (Sambrook and Russell 2006). GBS detection was performed by polymerase chain reaction (PCR) targeting the specific *cfb* gene (Ke et al. 2000).

All GBS-positive cultures were streaked on Columbia agar plates supplemented with 5% defibrinated sheep blood. After a 24-hour incubation period, plates were examined for colonies suggestive of GBS. Presumptive identification was performed based on colony morphology, Gram staining, and catalase test. All presumptive isolates underwent genomic DNA extraction using the phenol–chloroform method and were confirmed by PCR targeting the *cfb* gene. Subsequently, isolates were further validated through 16 S rRNA gene sequencing, following previously described protocols (Arruda et al. 2017).

### Characterization of GBS strains

Molecular capsular typing was performed using a multiplex PCR assay conducted in triplicate, as previously described (Imperi et al. 2010).

Antimicrobial susceptibility testing was performed using the disk diffusion method and interpreted according to the Clinical and Laboratory Standards Institute (CLSI) guidelines. The antibiotics tested included penicillin (10 µg), ampicillin (10 µg), chloramphenicol (30 µg), levofloxacin (5 µg), vancomycin (30 µg), tetracycline (30 µg), azithromycin (15 µg), erythromycin (15 µg), and clindamycin (2 µg) (all from CECON, São Paulo, Brazil) (Clinical and Laboratory Standards Institute 2022). Isolates classified as intermediate or resistant were grouped under the category non-susceptible (NS). MDR isolates were defined as those exhibiting non-susceptibility to at least one antimicrobial agent in three or more distinct antimicrobial classes (Magiorakos et al. 2012).

Macrolide–lincosamide–streptogramin (MLS) resistance phenotypes were evaluated using a double-disk diffusion assay (D test) with erythromycin and clindamycin. The M phenotype was defined as isolates resistant to erythromycin but susceptible to clindamycin, without inducible resistance in the zone of inhibition. The L phenotype was defined as isolates resistant only to clindamycin. Strains exhibiting the inducible MLS phenotype (iMLSB) showed resistance to erythromycin and susceptibility to clindamycin with a characteristic blunted D-shaped inhibition zone. Strains resistant to both erythromycin and clindamycin were classified as constitutive MLS phenotypes (cMLSB) (Clinical and Laboratory Standards Institute 2022).

Additionally, the presence of resistance genes was investigated using PCR assays. Genes associated with macrolide and lincosamide resistance (*ermA* (Seppälä et al. 1998), *ermB* (Klugman et al. 1998), and *mefA/E* (Sutcliffe et al. 1996)), as well as tetracycline resistance genes (*tetM* and *tetO*) (Trzcinski et al. 2000), were detected using specific primers in strains exhibiting non-susceptibility to these antibiotic classes.

### Statistical analysis

Continuous variables were summarized as means and ranges, and categorical variables as frequencies and percentages. Comparisons across study periods were performed using one-way analysis of variance (ANOVA) and chi-square or Fisher’s exact tests, as appropriate. Comparisons according to GBS colonization status were conducted using Welch’s *t* test and chi-square or Fisher’s exact tests. Multivariable logistic regression was used to identify factors independently associated with GBS colonization, with results reported as odds ratios and 95% confidence intervals. All statistical analyses and graphical outputs were performed in R (version 4.5.2; R Core Team, Vienna, Austria) using the tidyverse ecosystem for data manipulation and ggplot2 for figure generation.

## Results

Among 1,449 pregnant women, significant sociodemographic and clinical changes were observed across the pre-, during-, and post-COVID-19 periods, including differences in age, education, occupation, living area, vaginal discharge, and urinary tract infections (all *p* < 0.01) (Table 1). GBS was detected in 20.7% (300/1,449) of the pregnant women screened. The colonization rate showed variation across the study periods, ranging from: 22.6% before COVID-19 (BC), 21% during the pandemic (DC), and 19% after COVID-19 (AC) (Table 1). However, these differences were not statistically significant (*p* = 0.469).

**Table 1:** Sociodemographic and clinical characteristics of pregnant women by period

Aspects Evaluated	All (18 February to 23 November) *n* = 1449	Before COVID-19 (18 February to 20 February) *n* = 443	During COVID-19 (20 March to 23 April) *n* = 774	After COVID-19 (23 May to 23 November) *n* = 232	*p*-value
**Mean age**	28.2 (12–46 years old)	29.3 (14–46 years old)	29.2 (14–44 years old)	25.7 (12–43 years old)	< 0.001*
**Marital status**					
Married	76% (1091/1436)	73.2% (320/437)	79.4% (610/768)	69.7% (161/231)	0.003*
Single	24% (345/1436)	26.8% (117/437)	20.6% (158/768)	30.3% (70/231)	
**Ethnicity**					
Black	12.1% (175/1442)	10.7% (47/439)	13.6% (105/772)	10% (23/231)	0.200
Brown	72.8% (1050/1442)	71.8% (315/439)	72.5% (560/772)	75.8% (175/231)	
White	15.1% (217/1442)	17.5% (77/439)	13.9% (107/772)	14.3% (33/231)	
**Level of education**					
Illiterate	0.2% (3/1437)	0% (0/435)	0.3% (2/770)	0.6% (1/232)	< 0.001*
Basic education	17.2% (247/1437)	23.5% (102/435)	15.3% (118/770)	11.6% (27/232)	
High school	63.8% (917/1437)	62.5% (272/435)	64% (493/770)	65.5% (152/232)	
Higher education	18.8% (270/1437)	14% (61/435)	20.4% (157/770)	22.4% (52/232)	
**Occupation**					
Unemployed	8% (115/1431)	13.9% (60/433)	5.2% (40/766)	6.5% (15/232)	< 0.001*
Housewife	54.2% (776/1431)	55.9% (242/433)	53.4% (409/766)	53.9% (125/232)	
Employed	36.9% (528/1431)	27.5% (119/433)	41.4% (317/766)	39.7% (92/232)	
Student	0.8% (12/1431)	2.8% (12/433)	0% (0/766)	0% (0/232)	
**Living area**					
Urban/Peri urban	93.9% (1345/1433)	96.6% (420/435)	93.5% (716/766)	90.1% (209/232)	0.006*
Rural	6.1% (88/1433)	3.4% (15/435)	6.5% (50/766)	9.9% (23/232)	
**Clinical**					
Previous preterm delivery	7.3% (104/1434)	4.4% (18/438)	9.5% (73/766)	5.7% (13/230)	0.001*
Previous neonatal death	3.7% (53/1435)	3.4% (15/435)	3.9% (30/768)	3.4% (8/232)	0.900
Vaginal discharge	49.9% (715/1448)	55.5% (246/443)	51.2% (396/773)	31.5% (73/232)	< 0.001*
Urinary tract infections	53.6% (776/1449)	56.2% (249/443)	49.7% (385/774)	61.2% (142/232)	0.004*
GBS colonization	20.7% (300/1449)	22.6% (100/443)	21% (156/774)	19% (44/232)	0.469

Numbers in parentheses indicate the number of positive responses over the total number of participants who answered each variable. Denominators vary due to missing data for specific variables. An asterisk (*) indicates a statistically significant association (*p* < 0.05)

When stratified by colonization status, GBS-positive and GBS-negative women showed largely similar characteristics. Occupational status differed between groups, with a higher proportion of employed women among GBS-positive cases (*p* = 0.041). Vaginal discharge was significantly more frequent among GBS-positive women (*p* = 0.006), while no differences were observed for other variables (Table 2). In multivariable analysis, vaginal discharge was independently associated with increased odds of GBS colonization (OR: 1.46; 95% CI: 1.11–1.92; *p* = 0.006). Regarding occupation, housewives had significantly lower odds of GBS carriage (OR: 0.66; 95% CI: 0.50–0.88; *p* = 0.004). No associations were identified for other variables (Table 3).

**Table 2:** Sociodemographic and clinical characteristics according to GBS colonization

Aspects Evaluated	All Clinical Samples *n* = 1449	GBS + *n* = 300	GBS - *n* = 1149	*p*-value
Mean age	28.2 (12–46 years old)	28 (12–46 years old)	28 (14–46 years old)	0.788
**Marital status**				
Married	76% (1091/1436)	77.6% (232/299)	75.5% (859/1137)	0.510
Single	24% (345/1436)	22.4% (67/299)	24.5% (278/1137)	
**Ethnicity**				
Black	12.1% (175/1442)	11.7% (35/299)	12.2% (140/1137)	0.717
Brown	72.8% (1050/1442)	74.6% (223/299)	72.4% (827/1137)	
White	15.1% (217/1442)	13.7% (41/299)	15.4% (176/1137)	
**Level of education**				
Illiterate	0.2% (3/1437)	0% (0/299)	0.3% (3/1138)	0.587
Basic education	17.2% (247/1437)	18.1% (54/299)	17% (193/1138)	
High school	63.8% (917/1437)	65.2% (195/299)	63.4% (722/1138)	
Higher education	18.8% (270/1437)	16.7% (50/299)	19.3% (220/1138)	
**Occupation**				
Unemployed	8% (115/1431)	6.7% (20/298)	8.4% (95/1133)	0.041*
Housewife	54.2% (776/1431)	49.3% (147/298)	55.5% (629/1133)	
Employed	36.9% (528/1431)	43.6% (130/298)	35.1% (398/1133)	
Student	0.8% (12/1431)	0.3% (1/298)	1% (11/1133)	
**Living area**				
Urban	92.3% (1329/1433)	92.6% (275/297)	92.8% (1054/1136)	0.277
Peri urban	1.1% (16/1433)	0.3% (1/297)	1.3% (15/1136)	
Rural	6.1% (88/1433)	7.1% (21/297)	5.9% (67/1136)	
**Clinical**				
Previous preterm delivery	7.3% (104/1434)	7.7% (23/299)	7.1% (81/1135)	0.838
Previous neonatal death	3.7% (53/1435)	3% (9/298)	3.9% (44/1137)	0.603
Vaginal discharge	49.9% (715/1448)	56.7% (170/300)	47.5% (545/1148)	0.006*
Urinary tract infections	53.6% (776/1449)	55.5% (165/300)	53.2% (611/1149)	0.618

Numbers in parentheses indicate the number of positive responses over the total number of participants who answered each variable. Denominators vary due to missing data for specific variables. An asterisk (*) indicates a statistically significant association (*p* < 0.05)

**Table 3:** Factors independently associated with GBS colonization

Variables	OR	95% CI	*p*-value
**Age**	1.01	0.99, 1.03	0.4
**Period**			
Before COVID	—	—	
During COVID	0.90	0.66, 1.22	0.5
After COVID	0.76	0.49, 1.17	0.2
**Marital status**			
Married	—	—	
Single	0.95	0.69, 1.30	0.7
**Level of education**			
Basic education	—	—	
High school	0.94	0.66, 1.35	0.7
Higher education	0.79	0.50, 1.25	0.3
**Occupation**			
Unemployed	—	—	
Housewife	0.66	0.50, 0.88	0.004*
Employed	0.63	0.36, 1.06	0.091
**Living area**			
Peri urban	—	—	
Rural	5.05	0.91, 94.7	0.13
Urban	3.98	0.78, 72.6	0.2
**Vaginal discharge**	1.46	1.11, 1.92	0.006*
**Urinary tract infections**	0.99	0.75, 1.29	> 0.9
**Previous preterm delivery**	1.07	0.64, 1.72	0.8

*CI* - Confidence Interval, *OR -* Odds Ratio. ‘Illiterate’ and ‘Student’ categories excluded from the regression model. An asterisk (*) indicates a statistically significant association (*p* < 0.05)

**Fig. 1:**
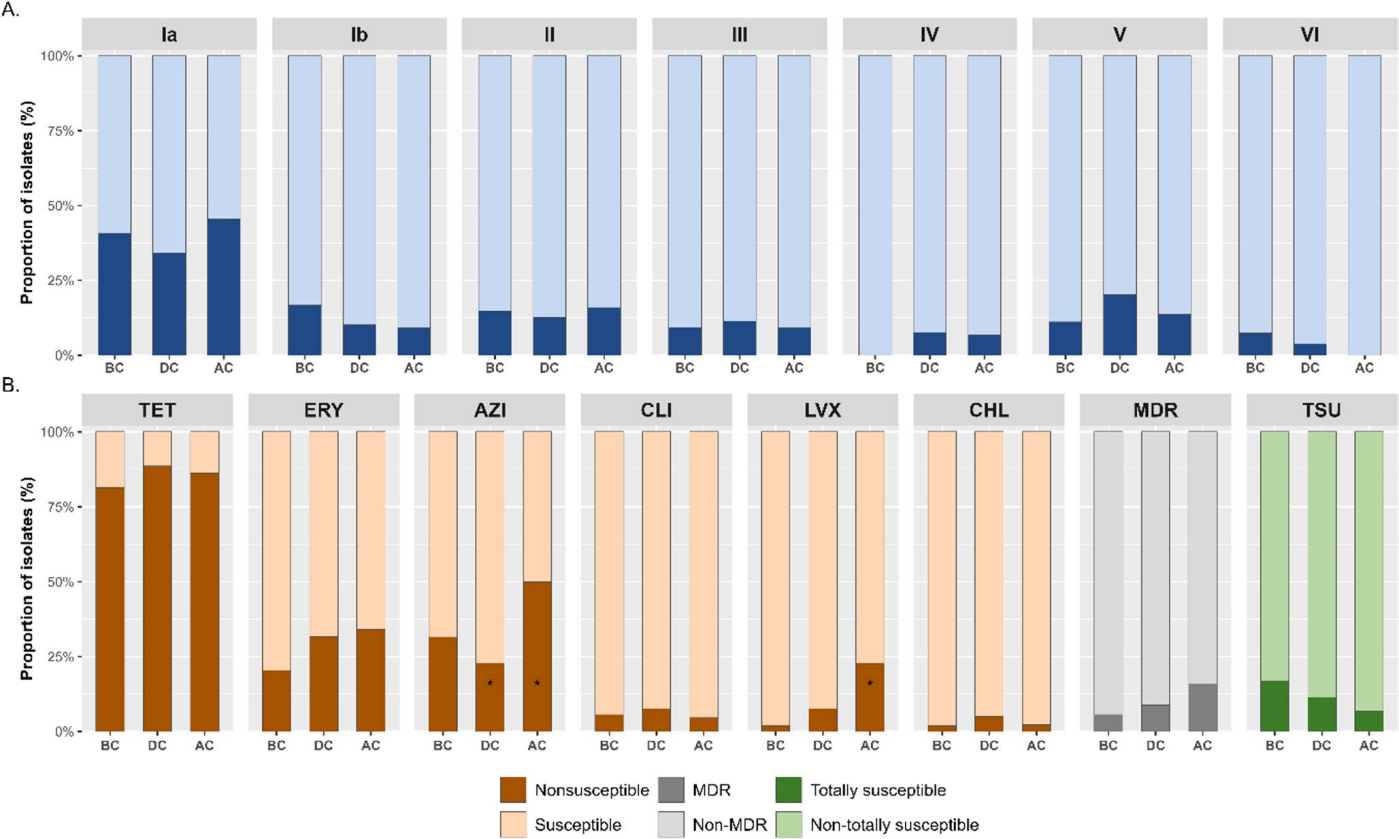
Distribution of serotypes (**A**) and antimicrobial resistance profiles (**B**) among Group B *Streptococcus* (GBS) isolates recovered from pregnant women in Brazil before, during, and after the COVID-19 pandemic. *Antibiotics:* TET – Tetracycline; ERY – Erythromycin; AZI – Azithromycin; CLI– Clindamycin; LVX – Levofloxacin; CHL – Chloramphenicol. *Additional categories:* MDR – Multidrug-resistant isolates; TSU – Totally susceptible isolates; Non-MDR – Non-multidrug-resistant isolates; Non-TSU – Non-totally susceptible isolates. *Time periods:* BC – Before the COVID-19 pandemic (February 2018 to February 2020); DC – During the COVID-19 pandemic (March 2020 to April 2023); AC – After the COVID-19 pandemic (May 2023 to November 2023). Statistical comparisons were performed using Fisher’s exact test. An asterisk (*) indicates a statistically significant association (*p *< 0.05) between the antimicrobial resistance phenotype and the corresponding serotype

Among colonized women, 177 isolates were available for characterization (54 BC, 79 DC, 44 AC). Overall, Serotype Ia was the most common (39%, 69), followed by serotypes V(15.8%, 28), II (14.1%, 25), Ib (11.9%, 21), III (10.2%, 18), IV (5.1%, 9), and VI (4%, 7). The serotype distribution varied across the periods without statistically significant differences. Detailed serotype proportions by period are presented in Fig. 1A.

All isolates were susceptible to penicillin, ampicillin, cefazolin, and vancomycin. Non-susceptibility was highest for tetracycline (85.4%, 152), azithromycin (32.2%, 57), erythromycin (28.8%, 51), levofloxacin (9.6%, 17), clindamycin (6.2%, 11), and chloramphenicol (3.4%, 6). A MDR profile was identified in 9.6% (17/177) of isolates, while 11.9% (21/177) were fully susceptible to all tested agents.

When stratified by period, non-susceptibility to erythromycin increased across BC, DC, and AC (20.4% → 31.6% → 34.1%). A non-linear pattern was observed for azithromycin (31.5% → 22.8% → 50%), while levofloxacin showed higher proportions in later periods (1.9% → 7.6% → 22.7%), with significant differences for both drugs (*p* = 0.0051 and *p* = 0.0018, respectively). Non-susceptibility to tetracycline remained consistently high, while clindamycin and chloramphenicol showed stable patterns across periods (Fig. 1B). Confidence intervals are presented in Supplementary Table S1.

**Fig. 2:**
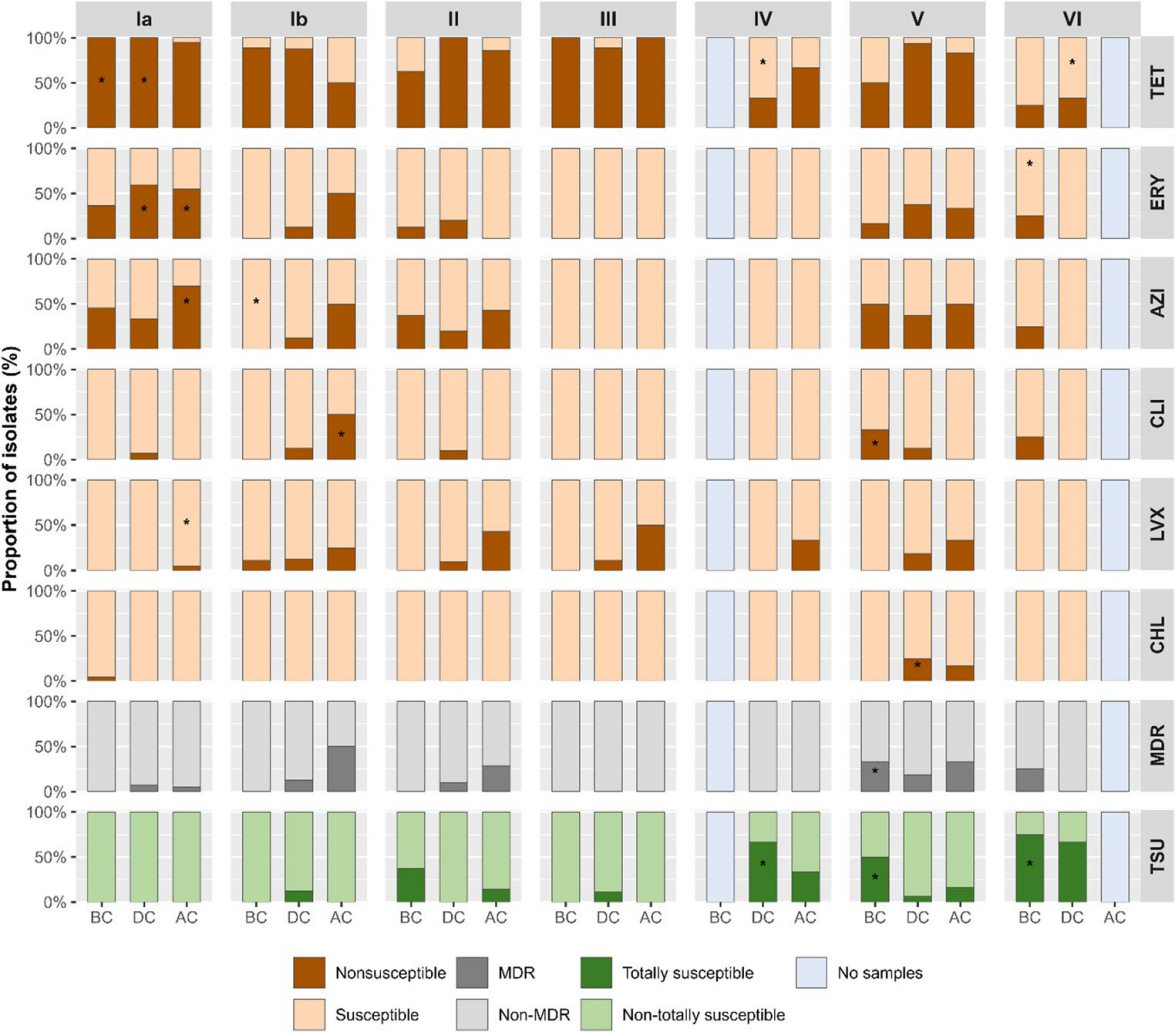
Distribution of antimicrobial susceptibility profiles among Group B *Streptococcus* (GBS) isolates recovered from pregnant women in Brazil, stratified by serotype and time period. *Antibiotics:* TET – Tetracycline; ERY – Erythromycin; AZI – Azithromycin; CLI– Clindamycin; LVX – Levofloxacin; CHL – Chloramphenicol. *Additional categories:* MDR – Multidrug-resistant isolates; TSU – Totally susceptible isolates; Non-MDR – Non-multidrug-resistant isolates; Non-TSU – Non-totally susceptible isolates. *Time periods:* BC – Before the COVID-19 pandemic (February 2018 to February 2020); DC – During the COVID-19 pandemic (March 2020 to April 2023); AC – After the COVID-19 pandemic (May 2023 to November 2023). Statistical comparisons were performed using Fisher’s exact test. An asterisk (*) indicates a statistically significant association (*p* < 0.05) between the antimicrobial resistance phenotype and the corresponding serotype

Serotype-specific resistance profiles showed marked heterogeneity (Fig. 2), with serotypes V, II, Ib, and Ia exhibiting the broadest non-susceptibility patterns. Tetracycline resistance remained high across most serotypes. Macrolide non-susceptibility (erythromycin and azithromycin) increased primarily in Ia, Ib, II, and V. Levofloxacin resistance, initially restricted to Ib in the pre-pandemic period, expanded to Ia, II, III, IV, and V in later periods. MDR increased over time in Ib, II, and V, while the proportion of fully susceptible isolates decreased in II, IV, and V.

**Fig. 3:**
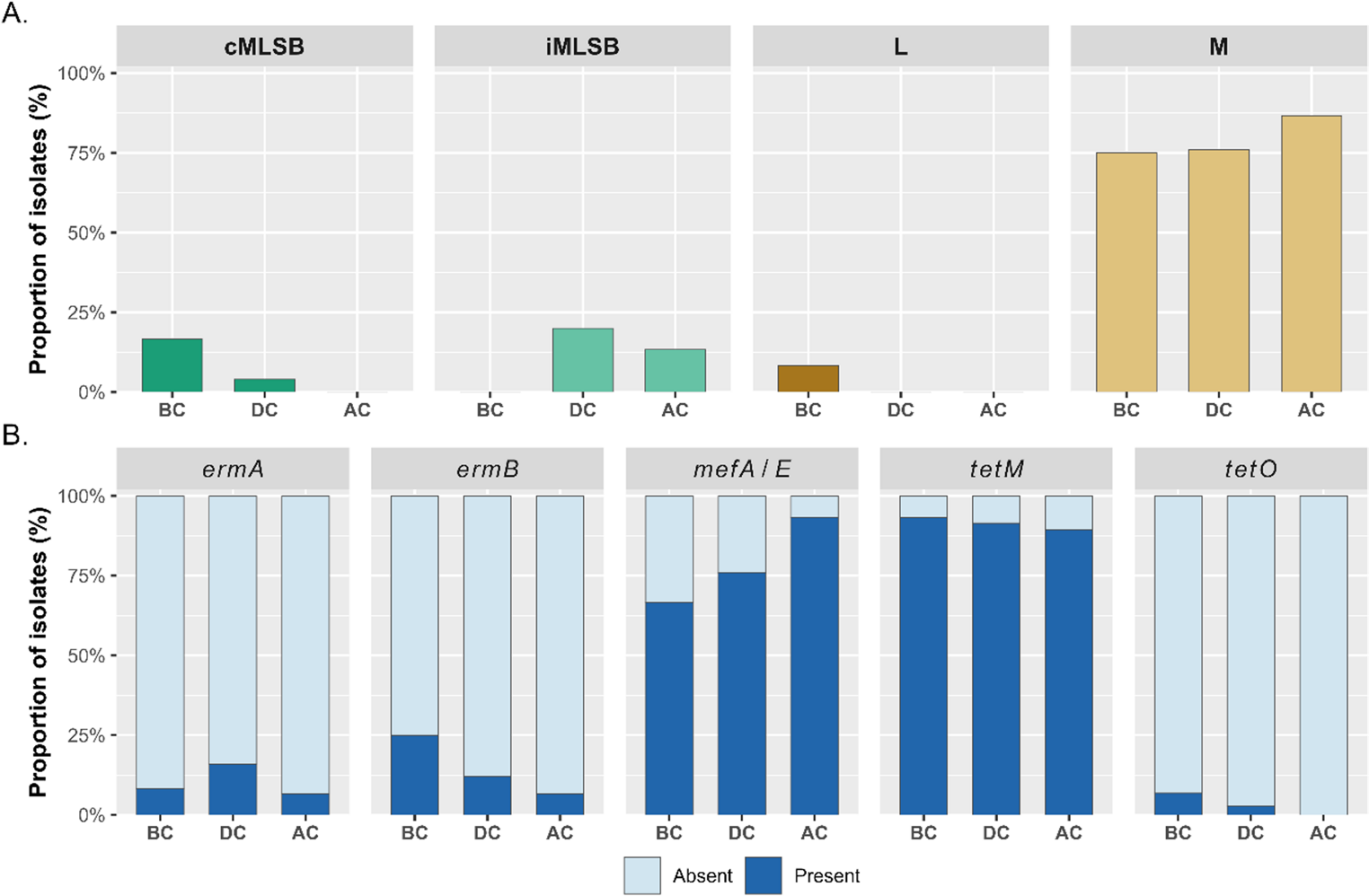
Distribution of MLSB resistance phenotypes and antimicrobial resistance genes among GBS isolates from pregnant women in Brazil, stratified by epidemiological period. **(A) ***Macrolide–Lincosamide–Streptogramin B* (MLSB) phenotypic resistance patterns. Phenotypes include: cMLSB– Constitutive MLSB resistance, iMLSB – Inducible MLSB resistance, L – Lincosamide resistance only, M – Macrolide resistance mediated by efflux Bars represent the proportion of isolates showing each phenotype within each epidemiological period: BC – Before COVID-19 pandemic (2018–2019), DC – During COVID-19 pandemic (2020–2022), AC – After COVID-19 pandemic (2023). **(B)** Distribution of antimicrobial resistance genes. Bars indicate proportions of isolates testing Present or Absent for each gene in each period (BC, DC, AC). No statistical significance testing was performed for this figure; values represent descriptive proportions

Among isolates non-susceptible to erythromycin and/or clindamycin (52/177; 29.4%), the M phenotype predominated (41/52; 82.4%) was consistently the most frequent across periods (Fig. 3A). The iMLSB phenotype (7/52; 13.5%) appeared only during and after the pandemic, whereas the cMLSB phenotype (3/52; 5.8%) decreased over time. The L phenotype (1/52; 1.9%) was detected only in BC (Fig. 3A).

Among the 52 macrolide- and/or lincosamide-non-susceptible isolates, *mefA/E* was the most frequent resistance gene (41/52; 78.8%), consistent with the predominance of the M phenotype. The *ermB* gene showed a decreasing trend (25% → 12% → 6.7%), while *ermA* showed no consistent pattern (8.3% → 16% → 6.7%) (Fig. 3B).

Among tetracycline-non-susceptible isolates (151/177; 85.7%), *tetM* was the dominant gene (138/151; 91.4%) and remained consistently prevalent across periods (93.2% → 91.3% → 89.5%), whereas *tetO* was rare (5/151; 3.3%) %) and was absent in the post-pandemic period (6.8% → 2.9% → 0) (Fig. 3B).

## Discussion

The COVID-19 pandemic markedly altered social behavior and healthcare utilization, creating a unique context to evaluate potential changes in maternal GBS epidemiology. In this study, colonization, serotype distribution, and antimicrobial resistance were evaluated across pre-, during-, and post-pandemic periods.

This investigation documented an overall GBS colonization rate of 20.7%, within the range reported in Brazil (4.2%–28.4%) and globally (17%–19%) (Russell et al. 2017, Nascimento et al. 2019). Colonization varied across the evaluated periods, ranging from 22.6% before the COVID-19 pandemic to 19% in the post-pandemic period. However, these differences were not statistically significant, indicating that temporal variation alone does not fully explain changes in colonization prevalence. The coexistence of shifts in sociodemographic and clinical characteristics across periods and stable GBS prevalence suggests that, in this setting, any potential effect of pandemic mitigation measures may have been counterbalanced by modifications in the population profile, resulting in attenuation of the observed decline. This, in turn, suggests resilience of GBS colonization dynamics despite population-level changes and reinforces the multifactorial nature of GBS colonization, likely shaped by interacting epidemiological and healthcare-related factors.

Other Brazilian studies have reported significant reductions in GBS detection during and after the pandemic, often attributed to mitigation measures such as improved hygiene and social distancing (Costa et al. 2022, Gomes et al. 2026). The divergence between our findings and those reported in other Brazilian regions suggests that temporal trends in GBS colonization may be context-dependent and influenced by regional epidemiological dynamics, healthcare access, and population characteristics.

Seven capsular serotypes (Ia, Ib, II, III, IV, V, and VI) were identified among the GBS isolates. Globally, serotypes Ia, Ib, II, III, and V account for most maternal colonization and neonatal disease, whereas serotypes VI–IX are more frequently reported in Asia (Russell et al. 2017, Furfaro et al. 2018, Madrid et al. 2017). In Brazil, studies consistently report serotype Ia as the most prevalent (Nascimento et al. 2019, Dutra et al. 2014, Carvalho et al. 2025). Our study also demonstrated that serotype distribution remained stable across periods, with minor fluctuations, suggesting subtle but ongoing dynamics and reinforcing the need for continuous surveillance. In addition, the observed serotype profile has direct implications for maternal GBS vaccines currently in late-stage development, particularly the hexavalent formulation (Ia, Ib, II, III, IV, and V) (Madhi et al. 2023). The high prevalence of vaccine-covered serotypes indicates substantial expected coverage. However, the detection of serotype VI highlights the need for post-vaccine surveillance (Furfaro et al. 2018, Seale et al. 2019, Khan et al. 2022, Trotter et al. 2023).

Rising antimicrobial resistance in GBS is a growing global concern. In this study, all isolates remained susceptible to β-lactams, consistent with Brazilian reports and supporting their continued use for intrapartum prophylaxis (Nascimento et al. 2019, Dutra et al. 2014, Barros 2021). In contrast, non-susceptibility to erythromycin, azithromycin, and levofloxacin increased substantially. Similar trends have been reported in Brazil, with rising erythromycin resistance and stable clindamycin and tetracycline resistance over time (Costa et al. 2022, Gomes et al. 2026). These findings align with global patterns of increased antimicrobial resistance during and after the COVID-19 pandemic, including reports from China showing marked increases in erythromycin and levofloxacin resistance (Wang et al. 2023). Clindamycin non-susceptibility remained low (6.2%) and stable, consistent with national data and lower than rates reported in the United States, Europe, and Asia (Bostanghadiri et al. 2025, Barros 2021, Francois Watkins et al. 2019). Tetracycline resistance remained high (85.4%), as expected, consistent with its widespread presence in GBS globally (Gergova et al. 2024, Bostanghadiri et al. 2025).

The M phenotype predominated and increased over time, alongside a decline in cMLSB and the emergence of iMLSB phenotypes during and after the pandemic. Genotypically, this pattern was accompanied by increased detection of *mefA/E* and reduced *ermB*, with *ermA* emerging during the pandemic, suggesting selective pressure toward efflux-mediated resistance. Similar findings have been reported by Barros et al. (Barros et al. 2024). For tetracyclines, *tetM* remained the most frequent determinant despite a declining trend, which was also observed for *tetO* (Gergova et al. 2024, Bostanghadiri et al. 2025).

The increase in MDR isolates, alongside the decline in fully susceptible isolates, indicates a shift in the GBS resistance landscape over time. This pattern is consistent with pandemic-related selective pressures, including widespread antimicrobial use. In Brazil, extensive community-level consumption of azithromycin during the COVID-19 pandemic, as well as other antimicrobials, has been well documented (Furlan and Caramelli 2021, Lalwani et al. 2021, Massarine et al. 2023), and we hypothesize that this may have favored the preferential expansion of resistant GBS lineages, even as overall colonization exhibited a numerical decline. Azithromycin non-susceptibility showed a non-linear pattern, contrasting with the steady increase observed for erythromycin. This does not support a direct association with antimicrobial use and likely reflects multifactorial influences, including changes in healthcare access, prescribing practices, circulating lineages, and isolate recovery. These findings should be interpreted cautiously, as individual-level antibiotic exposure was not assessed. Overall, they highlight the complexity of resistance dynamics and reinforce the need for continuous, resistance-focused surveillance beyond colonization rates alone.

This study is limited by its single-region design, which may restrict generalizability. Additionally, incomplete recovery of isolates may have affected the characterization of serotypes and antimicrobial resistance. Variation in recovery rates across periods—partly due to differences in processing time and storage conditions—may have influenced temporal comparisons and introduced potential selection bias. Furthermore, the small number of isolates in the post-pandemic period limits the precision of resistance estimates, which should be interpreted with caution. Despite these limitations, the internal validity of the study is preserved, although findings should be carefully considered when assessing temporal trends.

Together, these findings demonstrate temporal variation in GBS colonization, serotype distribution, and antimicrobial resistance across pre-, during-, and post-pandemic periods. Colonization and serotype distribution remained stable, with preserved β-lactam susceptibility, whereas macrolide and levofloxacin non-susceptibility varied significantly and MDR increased in later periods. These patterns provide relevant epidemiological insights in the context of rising post-pandemic antimicrobial resistance. As maternal GBS vaccines approach licensure, region-specific data will be essential to anticipate vaccine impact and monitor shifts in serotype distribution and resistance. Ongoing integration of phenotypic, genotypic, and clinical surveillance will be critical to track and respond to the evolving epidemiology of GBS.

## Supplementary Information

Below is the link to the electronic supplementary material.

Supplementary Material 1 (DOCX 18.3 KB) 

## Data Availability

The data sets supporting the conclusions of this article are included within the article. The raw data can be made available to interested researchers by the authors of this article if requested.
