# Supplementary material for: Temporal trends in Group B Streptococcus colonization, serotype distribution, and antimicrobial resistance among pregnant women in the Brazilian Amazon across the COVID-19 pandemic, 2018–2023
Source: Int Microbiol. 2026 Apr 25;29(5):701–11. doi: 10.1007/s10123-026-00832-1 (PMC13260128; doi:10.1007/s10123-026-00832-1)
Supplement: Supplementary file 1 — Supplementary Material 1 (DOCX 18.3 KB) [file 10123_2026_832_MOESM1_ESM.docx]

**Supplementary Table S1.** Antimicrobial resistance rates across pre-pandemic, pandemic, and post-pandemic periods.

| Antibiotic | Period | Resistant isolates (n) | Total isolates (N) | Resistance (%) | 95% CI |
| --- | --- | --- | --- | --- | --- |
| Azithromycin | Pre-pandemic | 17 | 54 | 31.5 | 19.5–45.6 |
|  | Pandemic | 18 | 79 | 22.8 | 14.1–33.6 |
|  | Post-pandemic | 22 | 44 | 50.0 | 34.6–65.4 |
|  | Overall | 57 | 177 | 32.2 | 25.4–39.6 |
| Chloramphenicol | Pre-pandemic | 1 | 54 | 1.9 | 0.0–9.9 |
|  | Pandemic | 4 | 79 | 5.1 | 1.4–12.5 |
|  | Post-pandemic | 1 | 44 | 2.3 | 0.1–12.0 |
|  | Overall | 6 | 177 | 3.4 | 1.3–7.2 |
| Clindamycin | Pre-pandemic | 3 | 54 | 5.6 | 1.2–15.4 |
|  | Pandemic | 6 | 79 | 7.6 | 2.8–15.8 |
|  | Post-pandemic | 2 | 44 | 4.5 | 0.6–15.5 |
|  | Overall | 11 | 177 | 6.2 | 3.1–10.8 |
| Erythromycin | Pre-pandemic | 11 | 54 | 20.4 | 10.6–33.5 |
|  | Pandemic | 25 | 79 | 31.6 | 21.6–43.1 |
|  | Post-pandemic | 15 | 44 | 34.1 | 20.5–49.9 |
|  | Overall | 51 | 177 | 28.8 | 22.3–36.1 |
| Levofloxacin | Pre-pandemic | 1 | 54 | 1.9 | 0.0–9.9 |
|  | Pandemic | 6 | 79 | 7.6 | 2.8–15.8 |
|  | Post-pandemic | 10 | 44 | 22.7 | 11.5–37.8 |
|  | Overall | 17 | 177 | 9.6 | 5.7–14.9 |
| Tetracycline | Pre-pandemic | 44 | 54 | 81.5 | 68.6–90.7 |
|  | Pandemic | 70 | 79 | 88.6 | 79.5–94.7 |
|  | Post-pandemic | 38 | 44 | 86.4 | 72.6–94.8 |
|  | Overall | 152 | 177 | 85.9 | 79.9–90.6 |

Resistance is presented as the number of non-susceptible isolates (n) over the total number of isolates tested (N), with percentages and exact 95% confidence intervals (Clopper–Pearson method).
